# A pilot study of how the past, present, and future are represented in three-dimensional space

**DOI:** 10.3389/fpsyg.2023.1071917

**Published:** 2023-03-22

**Authors:** Yoshiko Yabe, Sachie Yamada

**Affiliations:** ^1^Department of Life Sciences, Graduate School of Arts and Sciences, The University of Tokyo, Meguro-Ku, Tokyo, Japan; ^2^School of Cultural and Social Studies, Tokai University, Hiratsuka-shi, Kanagawa, Japan

**Keywords:** spatial representation of time, perspective of time, circle test, spatial temporal association of response code, reference frame

## Abstract

Numerous studies have shown that the representation of temporal concepts is associated with spatial features such as position and size. In a conventional task called the “Circle Test (CT),” participants are asked to express the relative importance of the past, present, and future and to demonstrate relationships among them by drawing three circles representing the past, present, and future. Studies on various participants, including refugees, patients living with serious illnesses, and adolescents, have used it to understand the temporal perspectives of different test takers. On the other hand, several studies have suggested that concepts of time are represented in three-dimensional (3D) space. It is expected that temporal concepts of the past, present, and future could be recorded using a 3D drawing task. Here we created a 3D version of CT (the “Sphere Test [ST]”) to investigate the sagittal representation of time and to record the relative time importance and relatedness, allowing for the shielding relationships and the laws of perspective. We conducted experiments with university students to compare the results from the CT and the ST. Our results suggested that not all on-screen overlapping can be interpreted as representing a connection between two time zones in 3D space. We also found correlations between the chosen sizes of the three circles in the CT and ST, i.e., the on-screen sizes of the past and present circles were positively correlated. In contrast, we observed no correlation between the on-screen sizes of the future circles in the two tests. The alignment pattern along the sagittal axis showed different patterns from the horizontal and vertical axes. In conclusion, this study sheds new light on the third dimension of the spatial representation of time and may help us understand the relationship between temporal perspectives and other factors, including mental health.

## 1. Introduction

How an individual views one’s past, present, and future has been shown to be associated with human behaviors and mental health (Strathman et al., 1994; Routledge et al., 2013; Turner et al., 2013; Sedikides et al., 2015; Lyu and Huang, 2016; Li et al., 2019; Burzynska and Stolarski, 2020; Drew et al., 2020; Newman et al., 2020; Zhi et al., 2021; Dennis et al., 2022; Olin et al., 2022). Researchers who were interested in the relationships between mental health and time perspective have tried to describe the types of time perspectives in various ways. For example, in an exploratory study by Zimbardo and Boyd, time perspective was categorized into five factors: the Past-Negative factor, which reflects a negative and aversive view of the past; the Present-Hedonistic factor, which reflects a hedonistic attitude toward time and life; the Future factor, which reflects a general future orientation; the Past-Positive factor, which reflects a positive view of the past; and the Present-Fatalistic factor, which reflects a fatalistic, helpless, and hopeless attitude toward the future and life (Zimbardo and Boyd, 1999). The questionnaire “Zimbardo Time Perspective Inventory (ZTPI)” created by Zimbardo and Boyd has been used in previous studies that have investigated how the attitudes toward the past, present, and future vary in different populations and how those attitudes would affect human behaviors, as has been reviewed by Peng et al. (2021). The ability to maintain a balance among the five perspectives without being biased toward a specific time perspective has been suggested as a healthy attitude (Zimbardo and Boyd, 1999; Mooney et al., 2017; Wiberg et al., 2017) and even increases cognitive performance (Witowska and Zajenkowski, 2021). Recent studies have shown the underlying neural mechanisms of time perspectives (Chen et al., 2018; Chen and Feng, 2022).

It has been suggested that how a person perceives the relatedness and relative importance of the past, present, and future is reflected in the way he/she spatially represents a time series of the past, present, and future (Mello et al., 2013; Mello and Worrell, 2015). A common method to measure an individual’s time perspective is the “Circle Test (CT)” invented by Cottle (Cottle, 1967). In the original CT, an experimenter asks participants to draw three circles representing the past, present, and future with two steps. For the first step, participants are required to express the relative importance of the past, present, and future with the relative sizes of the circles. For the second step, they are instructed to express the relatedness of the past, present, and future by the relative positions of the circles.

In the 1980s, Beiser and his colleagues performed a series of studies using CT on refugees who relocated to Vancouver (Beiser, 1987, 2009; Beiser and Hyman, 1997). Following the original method created by Cottle (1967), Beiser et al. categorized the patterns of circle overlaps into three types (i.e., Projection, Continuity, and Atomic) and used them as a measure of time-relatedness. They also categorized the patterns of the sizes of circles into five distinct types (i.e., Optimism, Hope, Pragmatism, Equivalent, and Nostalgia) to measure time dominance (Beiser, 1987). It was found that the majority of refugees chose a less-overlapping (“Atomic”) time-relatedness pattern as well as time-dominance patterns featuring a large future circle (i.e., “Optimism” pattern) or large future and present circles (i.e., “Hope”). In contrast, non-refugee Vancouverites did not show any specific preferences. Beiser et al. also showed that people who chose the “Atomic” pattern reported significantly lower depression scores than others. Regarding time dominance, the “Nostalgia” pattern, in which the past looms over the present and future, was shown to be associated with depression. Since then, a number of studies have used the CT to measure time orientation in various groups of participants, including adolescents (Haldeman, 1992; Mello et al., 2013; Mello and Worrell, 2015; Moon and Mello, 2021), adolescents with or without substance use (Barnett et al., 2013; Finan et al., 2022), young migrants (Radjack et al., 2020), imprisoned men (Sekulak et al., 2022), and patients with serious diseases such as cancer (Rovers et al., 2019) and brain tumors (Shigemune et al., 2021). Those studies that used CT to examine people experiencing disruptive events impacting their lives have suggested that there is a link between mental health and spatially expressed temporal orientation.

Although CT was originally designed as an instrument to measure how each participant feels the importance of time frames and connectedness between them, CT can also be used to record how each participant maps temporal concepts in a two-dimensional (2D) space. Leone et al. conducted CT on Spanish speakers and showed that the past, present, and future circles were located from left to right in most participants (Leone et al., 2018). Their findings are consistent with other studies that measured spatial representation of time using other tasks. Previous studies in which participants were required to place labels with time-related words have shown that people who write from left to right tend to associate the progress of time as proceeding from left to right (Woodin and Winter, 2018) while people who write from right to left tend to associate the progress of time as proceeding from right to left (Tversky et al., 1991). Among those who read from top to bottom, there is a tendency to associate earlier times with the topmost position and later times with the bottom (Bergen and Chan Lau, 2012). The mental image of time would not be only like a straight line but a circle or a spiral when participants were asked to graphically represent months of a year (Laeng and Hofseth, 2019). Each culture has a preferred format in which temporal sequences can be expressed spatially; these include calendars and railway timetables. It has been suggested that the common format used by a culture may affect the spatial representation of time during development (Starr and Srinivasan, 2021).

One could ask the question of whether the time–space association is just a metaphor for a verbal description of ideas related to time series, or whether it affects diverse cognitive processes. The answer would be the latter. The time–space association has been observed to affect the performance of response time tasks. Ishihara et al. (2008) found that, when participants were asked to judge whether an auditory tone was presented earlier or later than they expected, the responses to early-onset timing with a left key were quicker than those to late-onset timing and vice versa. They called this horizontal bias “STEARC: spatial–temporal association of response codes (Ishihara et al., 2008).” The STEARC was found to be absent when participants were required to discriminate a non-temporal feature of targets, even when the mapping between the lateral keys and the target onsets was congruent (Mariconda et al., 2022). A study of Spanish and English speakers showed that reaction time was shortened when participants were required to respond to “future” words with their right hands and to “past” words with their left hands (Ouellet et al., 2010). Other researchers have suggested that a vertical bias also affects the responses (Dalmaso et al., 2022). He et al. (2018) showed that reaction time was shortened when a response cue was presented on the top-left part of a screen following the presentation of a priming word linked to the past and vice versa. They also showed that participants looked at the top-left part of the screen during a free viewing period following the presentation of a priming word linked to the past and vice versa (He et al., 2018). The bias of horizontal and vertical time–space associations was shown to affect saccadic onset, even when the participants did not have to judge the timings of stimuli (Hartmann et al., 2021). It has been reported by Saj et al. (2014) that French-speaking patients with left spatial neglect caused by right-brain damage show difficulty in representing events that fall to the left of their mental timeline (Saj et al., 2014).

Time-related concepts are represented along the sagittal axis too. When describing time, it is common in many cultures to use words associated with spatial representations, including “far,” “close,” “forward,” and “back,” etc. to construe timings like objects with physical entities distributed in the the three-dimensional (3D) space. Cross-linguistic studies have indicated that the earlier timing tends to be associated with the words meaning “front” and the later timing tends to be associated with the words meaning “behind” (Moore, 2011) although the spatial representation of the time frames of the past, present, and future could be varied in different languages and contexts (Yang et al., 2023). Psychometric studies have shown that response time decreases when the time-related cue and the sagittal position of the hand to respond (Walker et al., 2017), the arrow key to press (Teghil et al., 2021), or the back or forward direction of whole-body motion (Hartmann and Mast, 2012) is congruent. It remains unclear what information is expressed using each horizontal, vertical, or sagittal axis and combinations of the three axes. A study on Mandarin speakers suggested that the horizontal axis would be used with either of the other two axes to represent time while the other two axes would not coexist (Ding et al., 2020). Núñez and Cooperrider (2013) introduced three types of time-related concepts: D-time, S-time, and T-span (Núñez and Cooperrider, 2013). The timings that belong to the D-time series category are thought to be distributed relative to the deictic center “now” (e.g., “past” and “future”). In contrast, the timings that belong to the S-time series category are thought to describe sequences of timings such as “earlier than” and “later than.” The “T-span” refers to a duration of time. A previous study on English speakers showed that congruent lateral (left-earlier and right-later) gestures are more strongly associated with S-time expression, and congruent sagittal (back-past and front-future) gestures with D-time expression (Casasanto and Jasmin, 2012).

**Figure 1:**
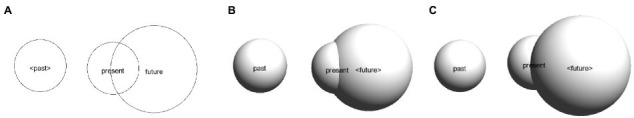
Examples of the screen images of CT **(A)** and ST **(B,C)**.

When considering the previous studies about the 3D representation of time mentioned above, it is expected that the time perspective from which an individual views one’s past, present, and future could be recorded in 3D space. Here, we created a “Sphere Test (ST)” in which participants expressed their spatial orientation of time in 3D space. We also conducted a preliminary trial on a population of healthy young Japanese speakers living in Japan to investigate the consistencies and differences between the information, which could be obtained using the ST and the CT. To illustrate the information that we would expect to obtain by adding the third axis, we present examples of the expressions of the past, present, and future in Figure 1. The three circles in Figure 1A display an example of the results from the original CT. In that figure, the present and future circles were attached to each other. The sizes of the past and present circles were smaller than the size of the future circle. How could those circles be expressed in 3D space if the participant tried to create a 3D image consistent with Figure 1A? One example is shown in Figure 1B, in which the present and future spheres were attached to each other. In addition, the sizes of the past and present spheres were smaller than the size of the future sphere again. Thus, the spatial features of Figures 1A,B have similar features on-screen and in 3D space. On the other hand, the 2D alignment of Figure 1A could be drawn with a 3D alignment different from Figure 1B. In Figure 1C, even though the on-screen sizes of the past and present spheres look virtually the same, the present sphere was as large as the future sphere in 3D space. Participants could use the attachment and shielding relationships to express the relative positions of the spheres and the perspective drawing to express the 2D sizes of them in the ST.

Please be aware that the example described above does not mean that the data obtained from the CTs in the previous studies contained ambiguities or missed some information. The instructions of the CT never asked participants to transfer the spheres in 3D space into the three circles in 2D space. In addition, of course, the CT has a significant advantage: it could be conducted using paper and pen, which was very important in decades past. In this study, inspired by the CT, we propose a computerized 3D task for future use as an interview tool in various groups of participants.

## 2. Materials and methods

### 2.1. Participants

A total of 32 graduate and undergraduate students at the University of Tokyo and Tokai University participated in this study. All proceedings were approved by the local ethics committees (i.e., Approval #794, the Ethical Review Committee for Experimental Research involving Human Subjects, The University of Tokyo and Approval #22086, the Ethical Review Committee for Experimental Research involving Human Subjects, Tokai University). The data from one participant was lost due to technical problems with a computer. Thus, the sample consisted of 31 participants (11 women, mean age = 20.61 years, range = 18–25). All participants were Japanese speakers who grew up in Japan. In the Japanese language, writing proceeds both from left to right and from top to bottom. Field studies have found that later time periods are mapped to the “back” and the earlier time periods to the “front” from an ego perspective while the past is mapped to the “back” and the future to the “front” from a field-based perspective (Moore, 2011). All participants were naïve to the purpose of the study.

### 2.2. Circle test

We computerized the conventional CT for this study. The task was programmed in MATLAB 9.10.0.1739362 (R2021a) using Psychtoolbox 3.0.18 (Brainard, 1997; Pelli, 1997; Kleiner et al., 2007). The size of the display was 345 mm × 194 mm. The pixel resolution of the screen was 1920 × 1080. All participants watched an instructional video clip just before starting the task. They were allowed to ask questions at any time during the task.

The first task in the CT was the size task. Five sample circles of different sizes—i.e., extra-small (30 pixels), small (60 pixels), medium (90 pixels), large (120 pixels), and extra-large (150 pixels) sizes—were presented in the row at the top. Each circle had a label indicating its size in the center. On the bottom row, three circles indicating the chosen sizes for the past, present, and future time zones were presented. Each of these had a label of the corresponding time zone in the center. Participants were instructed to choose one of the time zones by pressing a key. They were able to choose the “past” circle by pressing “k,” the “present” circle by pressing “g,” and the “future” circle by pressing “m.” The label on the chosen circle was presented with angle brackets. On the initial screen, the past circle was chosen. The participants were then instructed to choose the size of the chosen circle by pressing a number key so that the size expressed how important the time zone was for them. The initial sizes of the three circles were set as the medium.

The participants were then instructed to press “p” to start the position task. The three circles indicating the past, the present, and the future were presented in the chosen sizes. Again, participants chose one of the circles by pressing “k,” “g,” or “m.” They were required to manipulate the chosen circle *via* the drag-and-drop operation of a computer mouse so that the positions of the three circles expressed how the participants viewed the relationships between the past, the present, and the future.

Participants were able to return to the size task by pressing “s.” “A “press e to exit” prompt was presented once all sizes and positions were set. The final screen image was captured when “e” was pressed. An example of the circles generated by these tasks can be seen in Figure 1A.

### 2.3. Sphere test

Next, we modified the conventional CT to create the ST. For this task, participants were required to choose the relative sizes of three spheres to express the importance of past, present, and future and to arrange them in a virtual 3D space. This task was also programmed in MATLAB 9.10.0.1739362 (R2021a) using Psychtoolbox 3.0.18 and OpenGL 4.6. The renderer was NVIDIA GeForce GTX 1650 Ti/PCIe/SSE2 and the renderer driver version was 30.0.15.1289. The size of the display was 345 mm × 194 mm and the pixel resolution of this screen was 1920 × 1,080. The distance between the origin (*Z* = 0) of the OpenGL space coordinate and the virtual camera viewing the OpenGL space was 2.0 in the OpenGL unit. The objects in the OpenGL space were presented as images projected onto a plane, which was put at 1.9 units to the origin (i.e., *Z* = −1.9) of the OpenGL space. Objects closer than that plane or further away than 100 distance units (i.e., *Z* = 98) were clipped away. The camera was set upright, fixating the origin of the OpenGL space. The angle for the perspective projection was 25 degrees. Thus, the top-right and bottom-left positions on the plane at the depth of the origin of the OpenGL space were (0.79, 0.44, 0) and (−0.79, −0.44, 0). The top-right and bottom-left positions of the OpenGL frustum were (0.02, 0.04, −1.9) and (−0.02, −0.04, −1.9) on the near plane and (22.17, 39.41, 98) and (−22.17, −39.41, 98) on the far plane.

All participants watched an instructional video clip before starting the test. The instruction scenarios for the size and position tasks of the ST were the same as those for the CT. A new task called the “depth task” was added for the ST. Here, the participants were required to report how they viewed the relationships between the past, present, and future in a 3D space.

Five sample spheres (extra-small, small, medium, large, and extra-large sizes) and three spheres indicating the chosen sizes for past, present, and future time zones were presented at the start of the ST. The pixel sizes and positions of the spheres on the initial screen were the same as those for the initial screen of the CT. The initial depths were set to zero for all spheres. The participants were first required to complete the size and position tasks in the same way as described for the CT (see above). The participants were instructed to start the depth task by pressing “d.” They then pressed either the “k,” “g,” or “m” key to choose the past, present, or future, respectively, and moved the chosen sphere forward or backward by pressing the left or right mouse buttons. To make it easier for the participants to visualize the 3D locations of the spheres, we added a rotation function to the ST. During the position and depth tasks, participants were able to rotate the three spheres around the chosen sphere by pressing one of four arrow keys. Participants were also allowed to go back and forth among the size, position, and depth tasks by pressing “s,” “p,” and “d;” this could be repeated as many times as they desired. A “press e to exit” prompt was presented once all sizes, positions, and depths were set. The final screen image was captured when “e” was pressed. An example of the spheres generated by the ST is shown in Figures 1B,C.

### 2.4. Order of tasks

Written informed consent was obtained from each participant before beginning the experiment. The CT and ST trials were always run with the CT first and the ST second. All participants filled out a demographic questionnaire at the end of the session.

### 2.5. Analysis

Each data set obtained from a CT session consisted of chosen sizes and [*x*, *y*] positions for the past, present, and future circles. The “*x*” and “*y*” values recorded were the horizontal and vertical positions (expressed in pixels) relative to the bottom-left point of the display [0, 0]. Each of the chosen circle sizes also corresponded to a radius that could be expressed in pixels. Each data set obtained from an ST session consisted of chosen sizes and [*x*, *y*, *z*] positions expressed in the OpenGL space for the past, present, and future spheres. The “*x*,” “*y*,” and “*z*” values recorded were the horizontal, vertical, and sagittal positions in the OpenGL units.

We also calculated sizes in pixels and 2D [*x*, *y*] positions relative to the bottom-left point [0, 0] on the screen for each sphere. Next, the Euclidean distances between circles on the screen were calculated in pixels for each CT and ST. The Euclidean distances between spheres in the OpenGL space were calculated in the OpenGL unit for ST.

To investigate the consistency between the CT and the ST in the time-dominance domain, we conducted two types of statistical analysis. Firstly, we tested the similarity of the 2D (i.e., on-screen) sizes of the past, present, and future between the CT and the ST using correlation analysis. We obtained correlation coefficients that expressed the direction and magnitude of similarity between the sizes of circles from the CT and the 2D sizes of the spheres from the ST. We used a Bonferroni corrected αlevel of 0.017 (0.05 / 3) for these tests. Secondly, to test the similarity of the dominant time zones between the CT and the ST, we used an *Χ*^2^-test for consistency in a 2 × *K* table to determine the consistency in CT and ST results. The analysis examined the number of participants whose “past” circle or sphere was the largest of the three (i.e., “past-dominant”), the number of participants whose “present” circle or sphere was the largest of the three (i.e., “present-dominant”), the number of participants whose “future” circle or sphere was the largest of the three (i.e., “future-dominant”), the number of participants whose circles or spheres were the same size (i.e., “equivalent”), and the number of participants whose patterns of sizes differed from those listed above (“Other”).

Next, we focused on the 2D (i.e., on-screen) positions of three circles or spheres from the CT and the ST. Previous studies using 2D tasks have shown that past time zones tend to be represented on the left and future time zones on the right if participants generally read and write from left to right. It was expected that the circles or spheres would be distributed in different areas of the screen depending on the time zones. The effect of time zones on the positions of the three circles was analyzed for each axis using a linear mixed model (by REML) with participants included as a random effect. Tukey HSD tests were used for *post hoc* analyses. A Bonferroni-corrected α level of 0.025 (0.05 / 2) was used because this statistical test was repeated twice (the *X* and *Y* dimensions). We used the lmerTest and multicomp packages implemented in R version 4.1.1 for these analyses.

The positional information obtained from the ST consisted of three axes. We investigated the effect of time zones on the [*X*, *Y*, *Z*] position generated by ST. For this test, we used a Bonferroni-corrected α level of 0.017 (0.05 / 3) since this statistical test was repeated three times (i.e., once each for the *X*, *Y*, and *Z* dimensions). The standard deviations for each of the three axes were compared among the three time zones using Levene’s tests.

To test similarities between the two tests in the time-relatedness domain, we calculated the correlation coefficient between the CT and ST results with respect to the distance between the centers of the two circles or spheres. The significance of this correlation was then tested using a Bonferroni-corrected α level of 0.017 (0.05 / 3). Next, we focused on overlaps between a pair of circles or spheres. The distance between the surfaces of two circles or spheres was calculated by subtracting their radii from the distance between the centers of the two circles or spheres. A negative space value indicated that the two circles or spheres were attached.

## 3. Results

**Figure 2:**
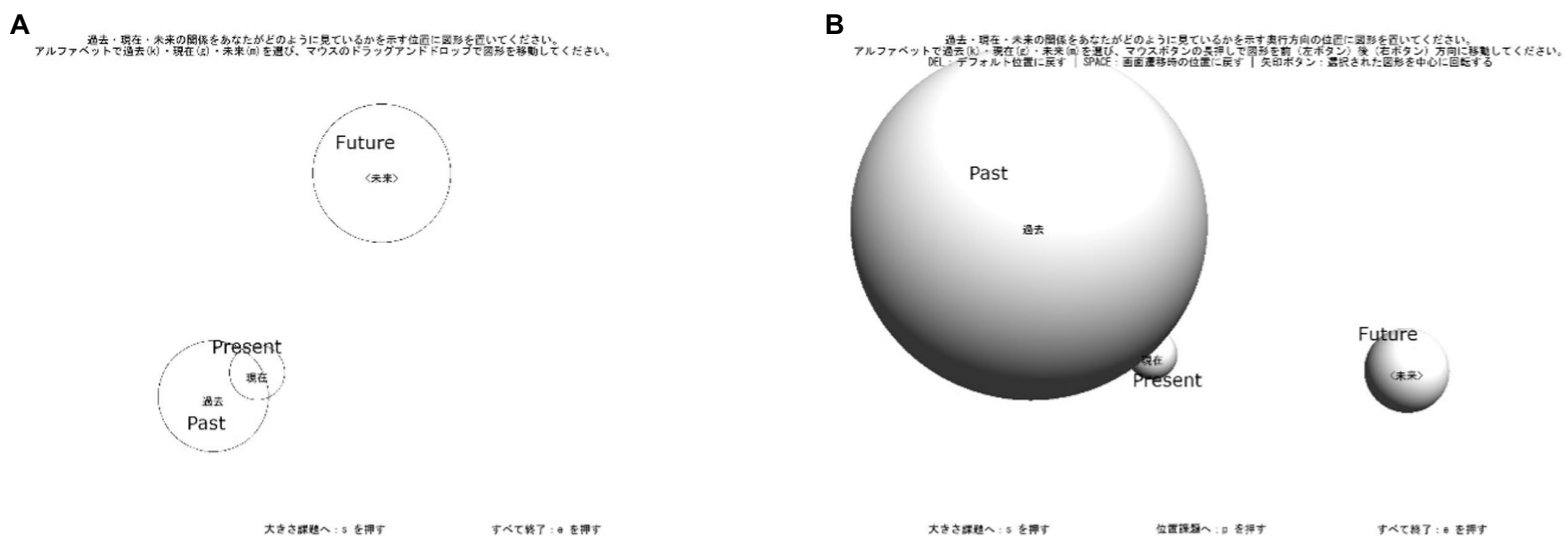
Examples of the screen images of CT **(A)** and ST **(B)** from a participant (ID: TFD0010). English descriptions were not present at the experiment.

Screenshot images taken at the end of the CTs and STs from all participants can be found in a public folder of the Open Science Forum.^1^ Examples of the screen-captured images from a participant are shown in Figure 2.

### 3.1. Time-dominance

**Figure 3:**
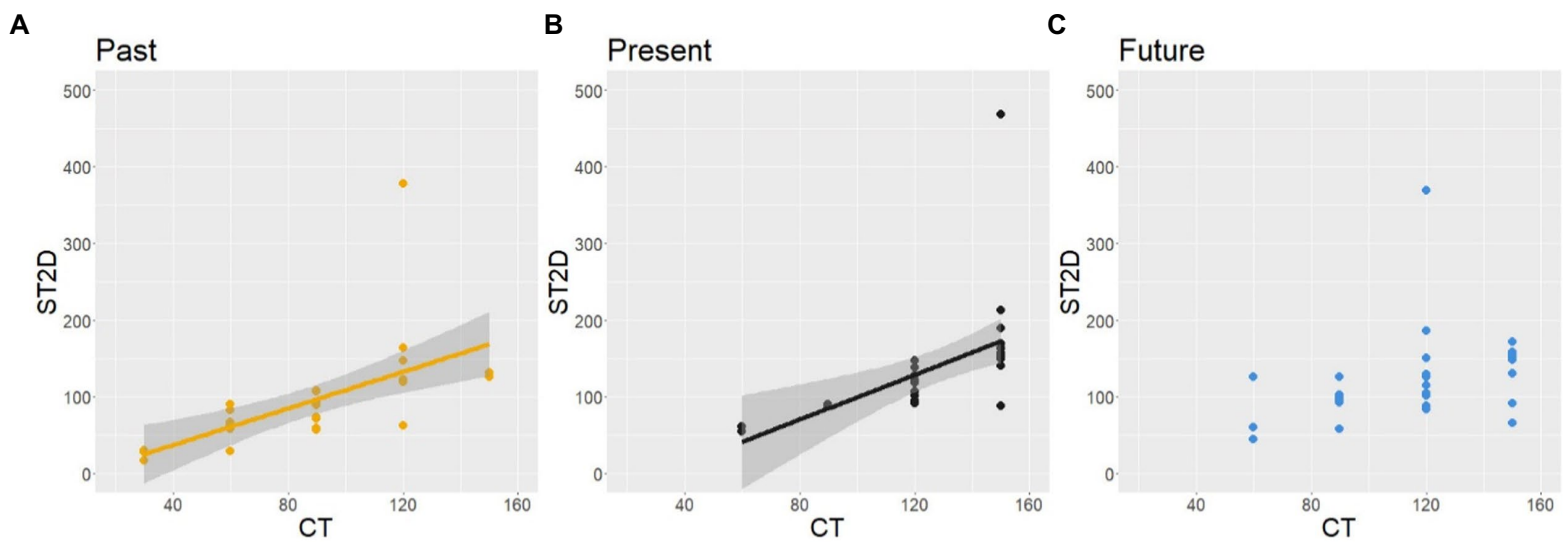
Sizes of spheres in ST on the screen as a function of the sizes of circles in CT. Each dot expresses a participant’s results for the past **(A)**, present **(B)**, and future **(C)**. The gray areas surrounding the regression lines indicate the confidence interval.

Firstly, we focused on the similarity between the CT and the ST with respect to the sizes of the three circles or spheres. The numbers of participants who chose the same sizes in the CT and the ST were 24, 27, and 25 for the past, present, and future circles or spheres, respectively. Significant correlations between the visual sizes in the CT and the ST were found for the past (*r* = 0.61, *p* < 0.001) and present (*r* = 0.55, *p* = 0.001), but not for the future (*r* = 0.37, *p* = 0.040). The on-screen sizes of circles in the CT and spheres in the ST are plotted in Figure 3. In summary, participants mostly chose similar sizes of circles or spheres in both tasks and drew circles and spheres of similar sizes on the screen.

Next, we focused on the order of the three sizes in the CT and the ST, since this has been used as a measure of “time dominance” in previous studies. Twenty-six out of 31 participants chose the same order of sizes in the CT and the ST. Table 1 shows the number of participants who chose the past, present, or future circles or spheres as the only largest. To test the consistency between the on-screen sizes in the CT and ST (see the ST 2D results shown in Table 1), we excluded the “Equivalent” and “Other” data where frequencies of on-screen sizes included zeros; this was done due to a limitation of the *Χ^2^*-test for consistency in a 2 × *K* table. The resulting test indicated that the dominant types were not different between the CT and ST on-screen results (*Χ*^2^ = 1.32, *p* = 0.516).

**Table 1:** Dominant sizes.

	Past-dominant	Present-dominant	Future-dominant	Equivalent	Other
CT	2	17	8	0	4
ST 2D	2	18	8	0	3
ST 3D	4	15	12	0	0

### 3.2. Position

**Figure 4:**
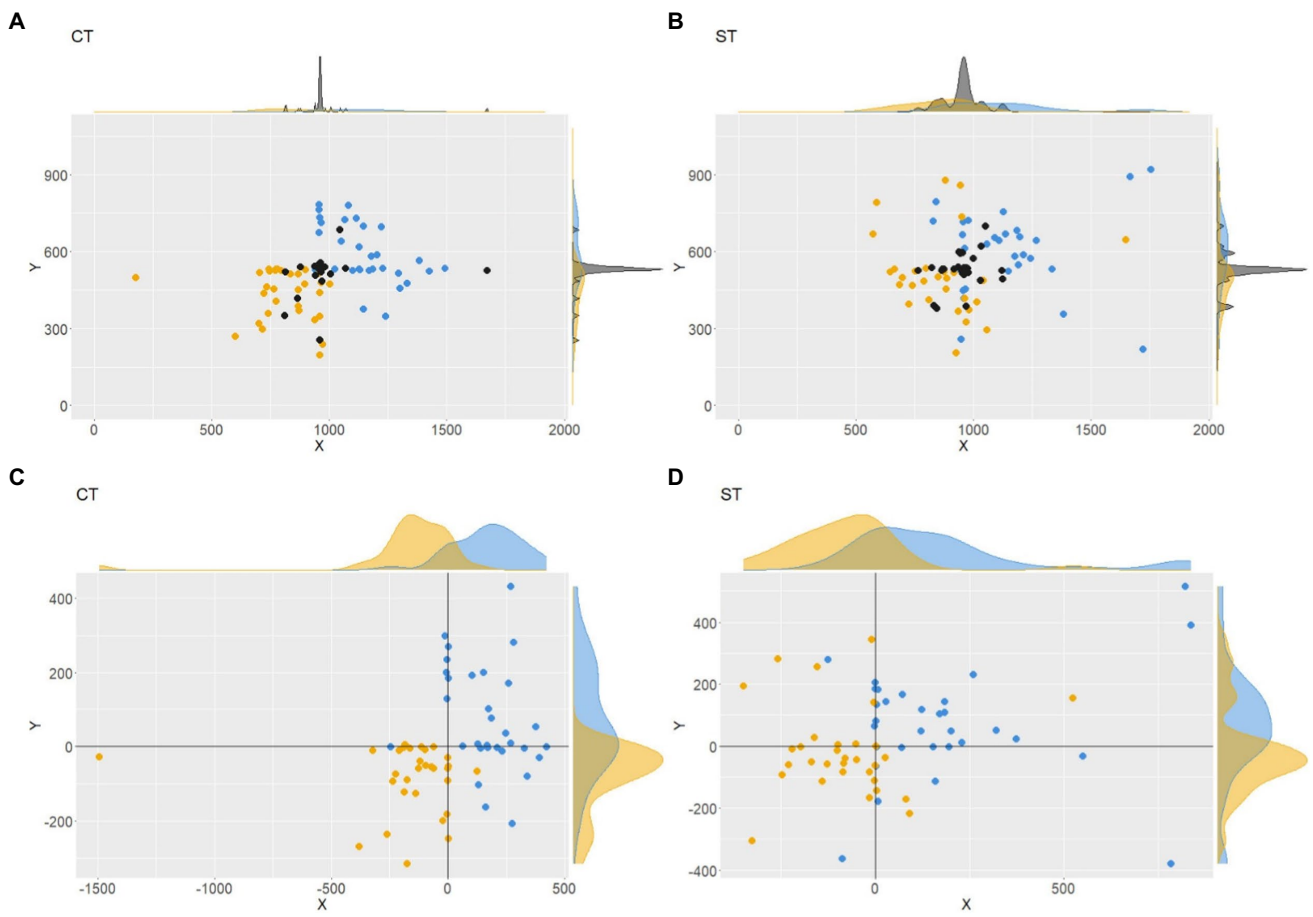
Absolute positions of circles in CT **(A)** and ST **(B)**. Each yellow, black, and blue dot expresses the past, present, or future position of a participant. The same results are plotted as positions relative to the present circle/sphere of the same participant for CT **(C)** and ST **(D)**.

In Figure 4A the yellow, black, and blue markers represent the positions of the past, present, and future circles from the CT, respectively. The mean and SD of the positions in the pixel are summarized in Table 2. The *X* values of the three circles were significantly different among time ranges in the CT (*F*(2, 90) = 39.926, *p* < 0.001). Moreover, *post hoc* tests revealed significant differences in the *X* values between the past and future circles (*z* = −8.94, *p* < 0.001), between the present and future circles (*z* = −4.50, *p* < 0.001), and between the present and past circles (*z* = 4.44, *p* = 0.003). *Y* values were also found to be significantly different among time zones in the CT (*F*(2, 90) = 20.62, *p* < 0.001). *Post hoc* tests revealed significant differences in *Y* values between the past and future circles (*z* = −6.42, *p* < 0.001), between the present and future circles (*z* = −2.98, *p* = 0.008), and between the present and past circles (*z* = 4.44, *p* = 0.002).

**Table 2:** Mean and STD values of *X* and *Y* positions.

			*X*			*Y*	
		Past	Present	Future	Past	Present	Future
CT	Mean	837.77	944.59	1138.32	433.73	515.68	615.27
	STD	101.33	57.24	149.24	86.78	46.41	117.1
ST	Mean	865.83	941.33	1093.61	485.03	518.68	598.55
	STD	132.89	80.62	203.26	144.46	62.48	145.66

Figure 4B shows the 2D (i.e., on-screen) positions of the past, present, and future spheres on the computer screen from the ST. The mean and SD of these positions are summarized in Table 3. The *X* values of the three spheres were significantly different among the time zones in the ST (*F*(2, 90) = 16.31, *p* < 0.001). *Post hoc* tests revealed significant differences in *X* values between the past and future spheres (*z* = −5.57, *p* < 0.001) and between the present and future spheres (*z* = −3.89, *p* < 0.001), but not between the present and past spheres (*z* = 1.68, *p* = 0.214). The *Y* values of the three spheres were not significantly different among time ranges either (*F*(2, 90) =3.50, *p* = 0.034). In summary, the time zones tended to be represented from the left to the right in 2D space (i.e., on the computer screen) both in the CT and the ST. The separation between the past and future, especially along the horizontal axis, can be seen more clearly when the positions of the past and future circles or spheres are plotted relative to the present (Figures 4C,D).

**Table 3:** Mean and STD values of *X*, *Y*, and *Z* positions.

		*X*			*Y*			*Z*	
	Past	Present	Future	Past	Present	Future	Past	Present	Future
Mean	−0.07	−0.012	0.108	−0.047	−0.013	0.048	0.09	0.199	−0.092
STD	0.102	0.061	0.153	0.096	0.041	0.108	0.523	0.355	0.673

**Figure 5:**
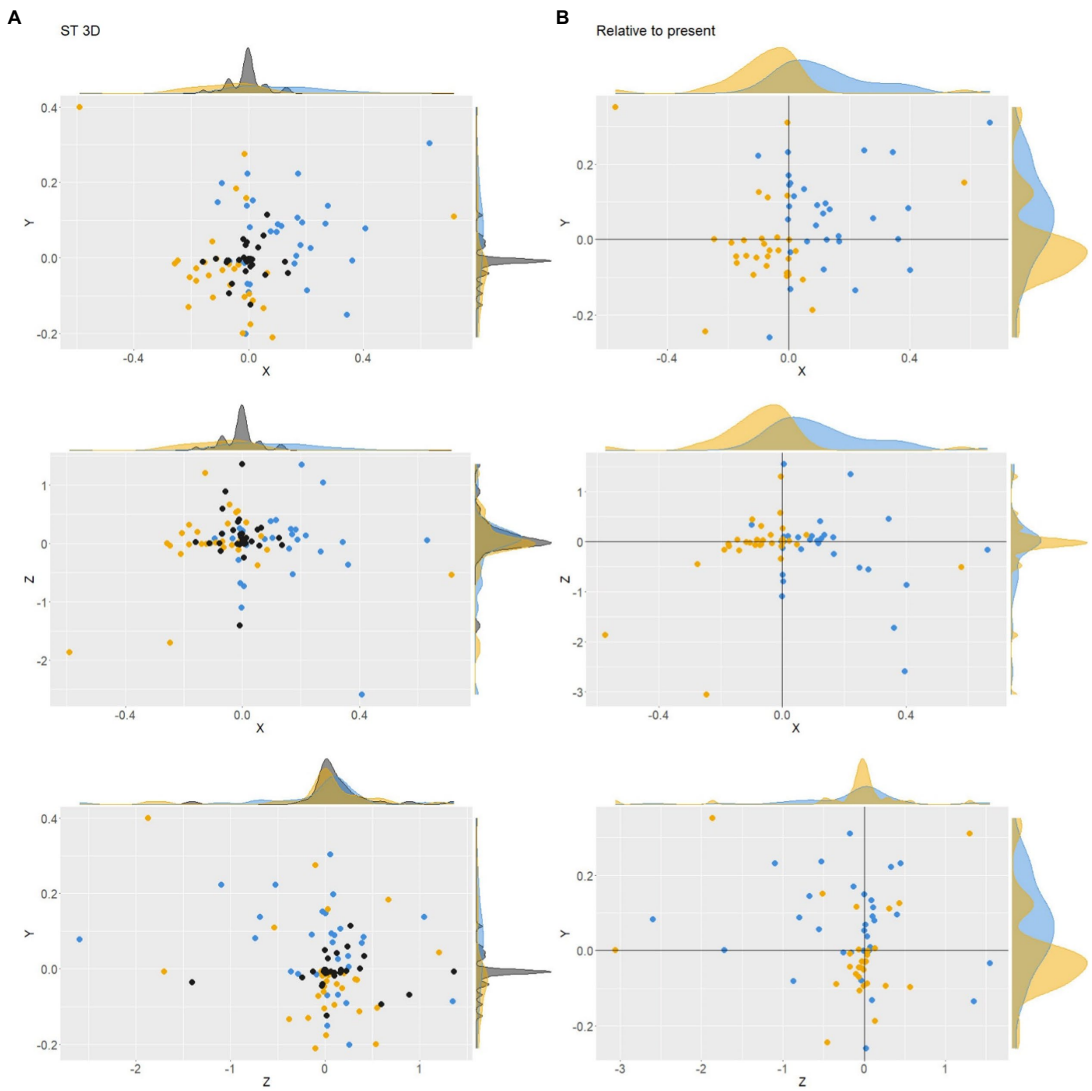
Absolute **(A)** and relative **(B)** positions in ST in the 3D space. Each yellow, black, and blue dot expresses the past, present, or future position of a participant.

We then investigated the differences among the positions of the three time zones for each of the three axes in the ST (Figure 5A). Dots with positive *X* values indicate that the sphere was located on the left. Dots with positive *Y* values indicate that the sphere was located on the upper part of the screen. Dots with small *Z* values indicate spheres located further away. The *X* values of the three spheres were significantly different among the time zones (*F*(2, 60) = 13.79, *p* < 0.001). *Post hoc* tests revealed significant differences in *X* values between the past and future spheres (*z* = −5.16, *p* < 0.001) and between the present and future spheres (*z* = −3.43, *p* = 0.001), but not between the present and past spheres (*z* = 1.72, *p* = 0.196). The *Y* and *Z*values of the three spheres were not significantly different among the time zones (*F*(2, 90) = 4.21, *p* = 0.018 for *Y*; *F*(2, 90) = 0.64, *p* = 0.529 for *Z*).

The density plots outside the scatter plot graphs show steep peaks on all axes of the present sphere. Levene’s test for homogeneity of variance showed that the *X* and *Y* positions were significantly different among the three spheres (*F*(2, 90) = 5.98, *p* = 0.003 for *X*; *F*(2, 90) = 7.06, *p* = 0.001 for *Y*). However, we found no significant difference in the variance of *Z* values (*F*(2, 90) = 0.70, *p* = 0.497). The positions of the past and future spheres relative to the present sphere are plotted in Figure 5B. Levene’s test for homogeneity of variance showed no significant difference between the three spheres for relative *X* positions (*F*(1, 60) = 0.44, *p* = 0.510), relative *Y* positions (*F*(1, 60) = 0.53, *p* = 0.468), and relative *Z* positions (*F*(1, 60) = 0.637, *p* = 0.428) axes.

**Figure 6:**
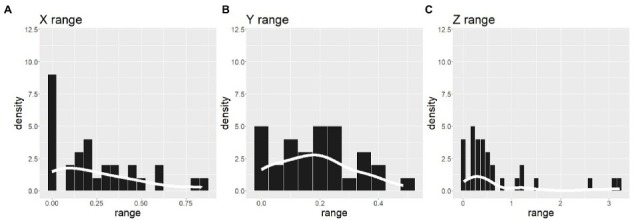
Frequencies of the positional ranges to draw spheres for *X* **(A)**, *Y* **(B)**, and *Z* **(C)** axes. The range values were different among a-c because of the OpenGL coordinate system.

**Figure 7:**
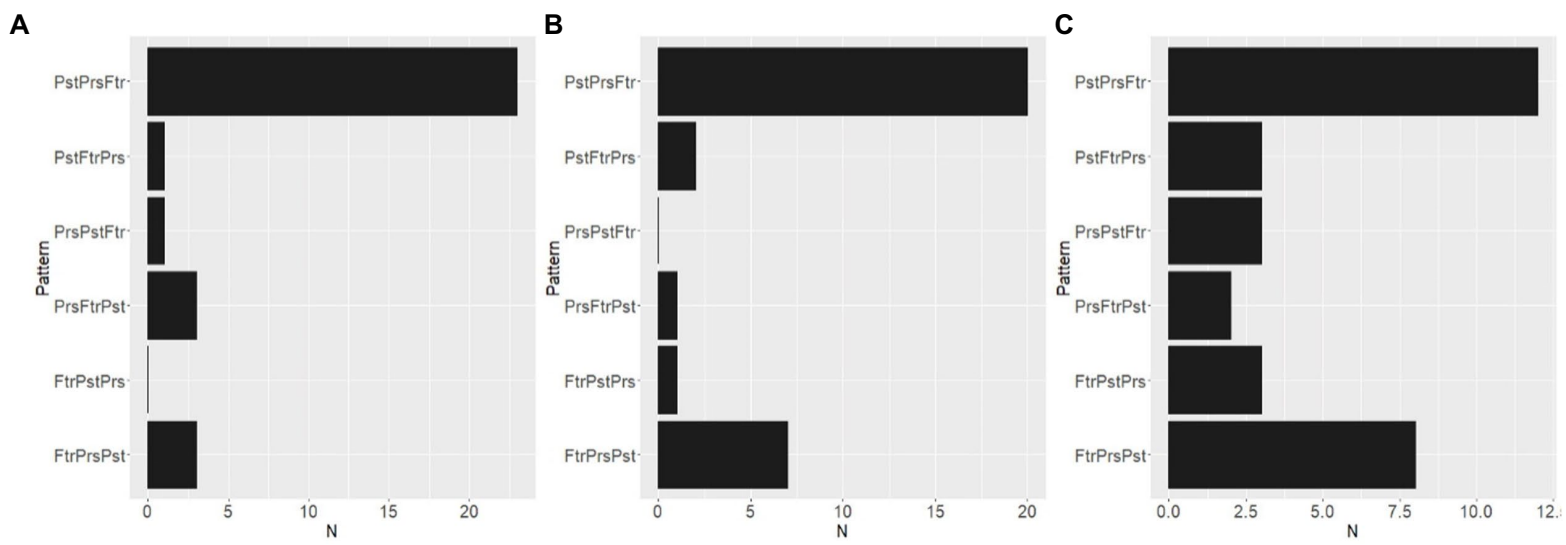
Frequencies at which participants drew the spheres from left to right **(A)**, from bottom to top **(B)**, or from the furthest to the nearest **(C)** in Past-Present-Future (“PstPrsFtr” in the figure), Past-Future-Present (“PstFtrPrs” in the figure), Present-Past-Future (“PrsPstFtr” in the figure), Present-Future-Past (“PrsFtrPst” in the figure), Future-Past-Present (“FtrPstPrs” in the figure), and Future-Present-Past (“FtrPrsPst” in the figure) orders.

The inter-participant pattern of the distribution of time zones described above showed no difference in how *Z* positions were used were used to draw the three spheres. Does this mean that the *Z* axis was not used in the ST? The histograms in Figure 6 show the frequencies of the range over which three spheres were distributed. The range was calculated by subtracting the minimum value from the maximum value for each axis and for each participant. It can be interpreted that the participant did not use the axis when the range for that axis was nearly zero. The mean and SD range values among participants were 0.25 ± 0.24 for *X*, 0.19 ± 0.13 for *Y*, and 0.69 ± 0.85 for *Z*. A linear mixed-effects model (Range ~ Axis with a random effect of the participants) with the R function lme revealed the significant effect of the axis on the range value (*F*(60, 60) = 10.45, *p* < 0.000). A *post hoc* Tukey comparison showed that the range value for the *Z* axis was larger than those for the *X* (*p* = 0.0007) and *Y* axis (*p *< 0.000). Thus, it is impossible to interpret the results of the ST in which the participant did not use the depth direction (*Z* axis) to express the time zones in 3D space. In addition, it should be noted that the range values along the *Z* axis were especially large in some participants. The skewness values were 0.87 for *X*, 0.25 for *Y*, and 1.84 for *Z*. The presence of participants who overly used the *Z* axis may indicate the potential importance of the *Z* axis. Figure 7A shows the numbers of participants whose spheres were put from left to right along the *X* axis in Past-Present-Future (“PstPrsFtr” in the figure), Past-Future-Present (“PstFtrPrs” in the figure), Present-Past-Future (“PrsPstFtr” in the figure), Present-Future-Past (“PrsFtrPst” in the figure), Future-Past-Present (“FtrPstPrs” in the figure), and Future-Present-Past (“FtrPrsPst” in the figure) orders. Most participants drew the three spheres in the Past-Future-Present order from left to right. This tendency is consistent with the previous studies, which have shown that past time zones tend to be represented on the left and future time zones on the right if the participants are familiar with reading and writing rightward. The numbers of participants with those alignment patterns along the *Y* axis (from bottom to top) are plotted in Figure 7B. Most participants chose to draw spheres in the Past-Present-Future order. However, approximately a quarter of participants chose the opposite order. The presence of those two patterns is consistent with the observations in previous studies. Finally, Figure 7C shows the frequencies of alignment patterns found along *Z* axes (from furthest to nearest). Although the Past-Present-Future order was observed most frequently, the difference between the frequency of the PastPresentFuture order and its opposite order was not as large as the differences found between opposite patterns along both *X* and *Y* axes. In addition, the frequencies of the other four patterns in which the time zones were not aligned according to the temporal order were chosen by approximately one-third of the participants.

### 3.3. Time-relatedness

**Figure 8:**
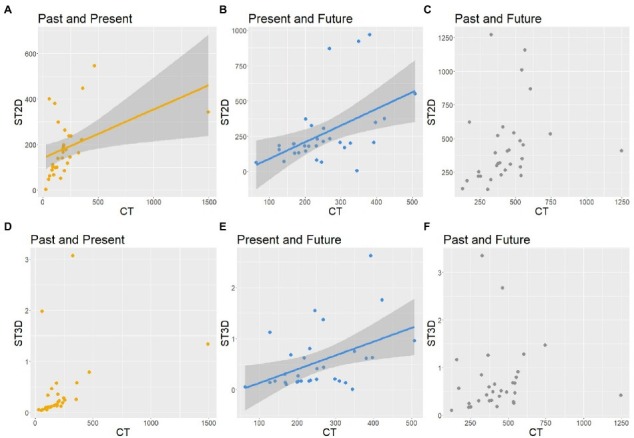
Distance between the past and present time zones in ST as a function of the distance in CT. Each dot expresses the past, present, and future positions of a participant. The gray areas surrounding the regression lines indicate the confidence interval. **(A)** Past-Present distance on the screen in ST. **(B)** Present-Future distance on the screen in ST. **(C)** Past-Future distance on the screen in ST. **(D)** Past-Present distance in 3D space in ST. **(E)** Present-Future distance in 3D space in ST. **(F)** Past-Future distance in 3D space in ST.

The distances between the centers of two circles from the CT and between the centers of two spheres on the screen from the ST are plotted in Figures 8A–C. Correlation analysis indicated that there was a significant correlation between the two tests in the distance between the past and present (*r* = 0.43, *p* = 0.016) and between the present and future (*r* = 0.48, *p* = 0.006). However, we found no correlation between the two tests in the past-future distance (*r* = 0.24, *p* = 0.018). As can be seen in Figures 8D–F, the distances in 3D space from the ST were not correlated with the distances in 2D space from the CT between the past and present (*r* = 0.36, *p* = 0.045) or between the past and future (*r* = 0.06, *p* = 0.769) but between the present and future (*r* = 0.45, *p* = 0.011).

**Figure 9:**
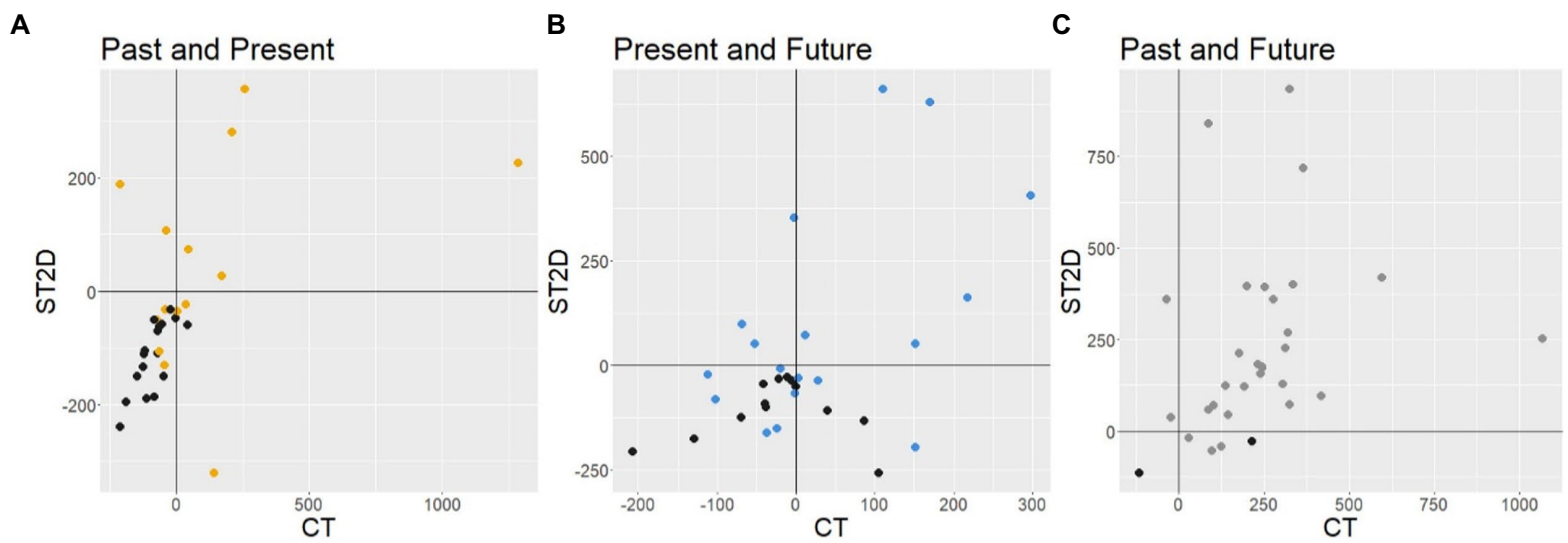
Distance between the surfaces of spheres in ST as a function of the distance between surfaces of circles in CT. Each dot expresses the past, present, and future positions of a participant. Black dots indicate that the two spheres are attached to each other in the 3D space in ST. **(A)** Past-Present distance on the screen in ST. **(B)** Present-Future distance on the screen in ST. **(C)** Past-Future distance on the screen in ST.

The distances between the surfaces of the circles and spheres are plotted in Figure 9. Each dot shows the distance between two spheres reported by a participant from the ST as a function of the distance between two circles from the CT reported by the same participant. Black dots indicate that the two spheres are attached to each other in the 3D space of the ST. The markers on the left relative to the vertical line (*x* = 0) of each graph indicate when two circles overlapped in the CT. The markers in the area below the horizontal line (*y* = 0) of each graph indicate when two spheres overlapped on the screen in the ST. Thus, a marker is plotted in the bottom-right area (*x* > 0 and *y* < 0) to show when a participant drew two time zones apart from each other in the CT and the same participant put those time zones overlapped in the ST. Especially for the present–future pairs (Figure 9B), we found that the overlap patterns were not consistent between the CT and ST. Some pairs of time zones that were not overlapped with each other in the CT (*x* > 0) were overlapping on the screen in the ST (y < 0). Some of the on-screen overlapping cases found in the ST (*y* < 0) were not attached (attached: black dots) but were rather shielding relationships in the 3D space.

## 4. Discussion

This study attempts to develop a new task, which we call the “Sphere Test,” based on a conventional 2D task (the “Circle Test”), to better understand how past, present, and future time zones are spatially represented in three dimensions. Data from healthy young people (*n* = 31) showed consistent results between the CT and the ST in general. We found that most participants chose the same sizes for both tasks, although the on-screen size of the future sphere in the ST did not correlate with the on-screen size of the future circle in the CT. The general patterns of the alignment of the circles or the spheres in the two tests were consistent with each other, too. The time zones are expressed from left to right in both 2D and 3D spaces in both the CT and ST tasks. We also found that the distance between a pair of circles, which indicates time-relatedness between the time zones, obtained from the CT mostly correlates with the 2D distance between pairs of spheres obtained from the ST.

Although the results obtained from the CT and ST are mostly alike, we found some inconsistencies between them. The lack of correlation between the on-screen sizes of the circles and the spheres representing the future time zone is an example of inconsistency. It can be seen that the 2D sizes of the future spheres (the vertical axis of Figure 3C) did not increase clearly when the sizes of the future circles increased from 120 pixels to 150 pixels. Some of the participants who chose extra-large sizes for the future would have put the future spheres far away from the viewpoint to show them smaller.

Another inconsistency was found in the relative positions of the past and present circles and the spheres. In this study, we found no significant differences between the *X* values of the past and present spheres in the ST (both in 2D-and 3D-space) despite the two facts that, firstly, the positions of the past and present circles were found to be different in the CT and that, secondly, we found a correlation between the on-screen distance between the past and present spheres in the ST and the distance between the past and present circles in the CT. This inconsistency would suggest that the absolute positions of the spheres in 3D space vary while the relationships among them are kept.

We also found that overlaps of circles or spheres in two dimensions could indicate “shielding,” i.e., a case in which two time zones were represented as completely apart when plotted in 3D space but appeared to be overlapping in 2D space. The depth dimension introduced by the ST may also affect the time-dominance expression by visual assessment of the relative sizes of the time zones. Although the on-screen sizes in the ST were shown to be correlated with those in the CT for the past and present time zones, the correlation between the future time zone sizes in the CT and the ST was not significant. The wider individual differences in *Z* values in the future spheres may reduce the degree of correlation between the visual sizes of future time zones in the CT and ST.

It is worth discussing the spatial order of the three spheres along the *Z* axis. Previous psychometric studies of spatial representation of deictic timing have suggested that the past and future are associated with the space behind and in front of us, respectively (Hartmann and Mast, 2012; Walker et al., 2017). Consistent with those previous studies, we found that the pattern of spatial alignment of the three spheres most chosen by participants was the Past-Present-Future order (the past sphere was the furthest). However, in the meantime, we found that the second most dominant pattern was the opposite order: the Future-Present-Past order. We would suggest two possible interpretations of why both of those two patterns became dominant. First, those two dominant patterns reflect the influence of the spatial expressions of time in the language that the participants spoke. Cross-lingual studies have shown that many languages use spatial metaphors to express time in the spatial reference frame (Moore, 2011, 2017; Tenbrink, 2011; Brown, 2012). Moore (2011) has suggested that the Japanese word “mae,” which means “front” in English, is used to describe an earlier time in a temporal sequence from the field-based reference frame and to describe the future from the egocentric reference frame. The participants who chose the Past-Present-Future order would have had the field-based time perspective in which the past was imagined to be located at the front end. On the other hand, the participants who chose the Future-Present-Past order would have viewed the time from an ego perspective, moving forward to the future. Another possible interpretation is that the *Z* values and, hence, the patterns along the *Z* axis showed the subjective distance between the participant and the time zones. The participants who plotted the past sphere nearest would have been nostalgic or regretful, whilst the participants who plotted the future sphere nearest would have been futuristic or anxious. We also found that the irregular patterns of sagittal (i.e., along the *Z* axis) orders (i.e., the Past-Future-Present, Present-Past-Future, Present-Future-Past, and Future-Past-Present) were chosen by approximately one-third of the participants. In contrast, the numbers of participants who chose those irregular orders for the lateral (i.e., along the *X* axis) and vertical (i.e., along the *Y* axis) orders were very low. The *Z* values from the ST may be speculated to express the individual differences in the temporal perspective of participants instead of the mere temporal progress from the past to the future.

This study has three limitations that must be mentioned. First, the sample size of this study is not big enough to validate the ST. The previous studies that aimed at validating the original CT recruited several 100 participants. Second, all the participants we recruited were university students who had grown up in Japan. It is impossible for us to say what the results of the ST would show on a global scale. The CT has become reliable by being used in the studies of various groups of participants. And finally, as the ST uses a 2D computer screen, it would be more difficult for the participants to understand the 3D relationships of the spheres in the ST than to understand the 2D relationships of the circles in the CT.

In summary, this pilot study suggests that the 3D ST task may provide mostly consistent information about the temporal perspective of participants with the 2D CT task. The advantage of the ST compared to the CT is that the expressions of spheres in 3D space make it possible to add the shielding and attach relationships of the spheres, information relating to which did not exist in the CT. In addition, the alignment pattern of the three spheres along the *Z* axis showed a different pattern from those along the *X* and *Y* axes. Future work is needed to examine how *Z* values of the spheres and the relationships between two spheres on the *Z* axis, including the overlapping and shielding relationships, are affected by various factors, such as language, culture, participant stage of life, and mental health.

## Data availability statement

The datasets presented in this study can be found in online repositories. The names of the repository/repositories and accession number(s) can be found at: https://osf.io/eadkj/.

## Ethics statement

The studies involving human participants were reviewed and approved by Ethical Review Committee for Experimental Research involving Human Subjects (The University of Tokyo), and the Ethical Review Committee for Experimental Research involving Human Subjects (Tokai University). The patients/participants provided their written informed consent to participate in this study.

## Author contributions

YY designed the study, collected and analyzed data, and wrote the manuscript. SY designed the study and wrote the manuscript. All authors contributed to the article and approved the submitted version.

## Funding

This study was supported by JSPS Grant-in-Aid for Challenging Research (Exploratory) grant #21K18574.

## Conflict of interest

The authors declare that the research was conducted in the absence of any commercial or financial relationships that could be construed as a potential conflict of interest.

## Publisher’s note

All claims expressed in this article are solely those of the authors and do not necessarily represent those of their affiliated organizations, or those of the publisher, the editors and the reviewers. Any product that may be evaluated in this article, or claim that may be made by its manufacturer, is not guaranteed or endorsed by the publisher.

## Footnotes

^1^ https://osf.io/eadkj/
